# ASSOCIATIONS BETWEEN HEART RATE AND PHYSICAL ACTIVITY IN PEOPLE WITH POST-COVID-19 CONDITION ACCOUNTING FOR MYALGIC ENCEPHALOMYELITIS/CHRONIC FATIGUE SYNDROME SYMPTOMS

**DOI:** 10.2340/jrm.v58.43340

**Published:** 2026-01-27

**Authors:** Rachel ADODO, Antonio SARMENTO DA NOBREGA, Rodrigo VILLAR, Sandra C. WEBBER, Diana C. SANCHEZ-RAMIREZ

**Affiliations:** 1 Department of Respiratory Therapy, College of Rehabilitation Sciences, University of Manitoba, Winnipeg; 2 Faculty of Kinesiology and Recreation Management, University of Manitoba, Winnipeg; 3 Department of Physical Therapy, College of Rehabilitation Sciences, University of Manitoba, Winnipeg, Canada

**Keywords:** long COVID, physical activity, heart rate, tachycardia

## Abstract

**Background:**

Tachycardia after mild activity or during rest is a common complaint among people with post-COVID-19 condition (PCC). Understanding the relationships between heart rate (HR) and physical activity (PA) in this population is crucial for developing appropriate rehabilitation protocols.

**Objective:**

To investigate the associations between HR and PA in individuals with PCC, accounting for Myalgic Encephalomyelitis/Chronic Fatigue Syndrome (ME/CFS) symptoms.

**Design:**

Observational study.

**Subjects:**

Sixteen adults with PCC (81% females, mean age 51 **±** 12 years).

**Methods:**

Participants were instructed to use 2 wearable devices (Garmin smartwatch and ActiGraph accelerometer) during waking hours over 4 days while performing daily activities. Average HR, percentage of time in tachycardia (time with HR > 100 bpm), and daily step count were assessed. The accelerometer counts per minute was used to categorize daily PA as sedentary, light intensity, and moderate-to-vigorous (MVPA).

**Results:**

Participants wore the watches and accelerometers for a mean of 11.36 **±** 2.60 and 12.51 **±** 1.94 h per day, respectively. Average daily HR increased with increasing PA levels from sedentary to MVPA. However, the percentage of time in tachycardia was significantly lower during periods of MVPA compared with sedentary periods, even after adjusting for ME/CFS symptoms.

**Conclusion:**

Individuals with PCC in our study experienced more tachycardia during periods of minimal physical activity compared with periods categorized as MVPA.

## LAY ABSTRACT

This study explored the associations between heart rate and physical activity in individuals with post-COVID-19 condition accounting for Myalgic Encephalomyelitis/Chronic Fatigue Syndrome symptoms. Tachycardia (elevated heart rate) during mild activity or at rest is common among those with post-COVID-19 condition, and understanding this relationship is important for developing effective rehabilitation strategies. The study involved 16 adults with post-COVID-19 condition (81% females, average age 51 years) who wore a Garmin smartwatch and an ActiGraph accelerometer for 4 days to track their heart rate and daily physical activity. The study found that heart rate increased with higher levels of physical activity, from sedentary to moderate-to-vigorous physical activity. However, the percentage of time spent in tachycardia was significantly lower during moderate-to-vigorous physical activity compared with sedentary periods, even after adjusting for Myalgic Encephalomyelitis/Chronic Fatigue Syndrome symptoms. The results suggest that individuals with post-COVID-19 condition experience more tachycardia when engaging in minimal physical activity, highlighting the importance of understanding heart rate-physical activity dynamics for better managing post-COVID-19 condition rehabilitation.

The Coronavirus disease 2019 (COVID-19) illness can persist beyond the initial stage and lead to post-COVID-19 condition (PCC), which is the continuation or development of symptoms 3 months after the initial infection and lasting at least 2 months (1). PCC is prevalent in about 20% of COVID-19 survivors worldwide, with a higher prevalence identified in female adults and those hospitalized during the acute infection (2). PCC symptoms are multisystemic (3) and may affect physical activity participation, work capacity, and social functioning (4). Therefore, appropriate interventions may be needed to help affected individuals recover and return to optimal functioning.

Individuals with PCC commonly report tachycardia with little to no activity (3), potentially due to autonomic nervous system dysfunction (5), which may be expressed as inappropriate sinus tachycardia or postural orthostatic tachycardia syndrome (POTS) (6). Inappropriate sinus tachycardia is marked by a disproportionate HR increase relative to physiological demand (7), whereas POTS is characterized by tachycardia upon standing (8). Additionally, some individuals with PCC may develop myalgic encephalomyelitis/chronic fatigue syndrome (ME/CFS) (9), which is the worsening of symptoms due to an atypical response (e.g., an abnormal loss of physical and mental stamina) after physical or mental exertion (10).

Evidence has shown that physical activity (PA) enhances well-being (11) and promotes physical, mental (12, 13), cardiovascular (14), and pulmonary health (15). However, these benefits may not directly apply to individuals with PCC (9, 10). The risk of post-exertional malaise in this population makes the use of established PA guidelines or exercise interventions uncertain, and the presence of dysautonomia may limit the use of heart rate (HR) as a reliable parameter for prescribing, guiding, and adjusting recommended PA. Consequently, the National Institute for Health and Care Excellence and the World Health Organization recommend caution when prescribing exercises for these individuals (16, 17).

The lack of understanding regarding the effects of PA in individuals with PCC, together with the reported cardiorespiratory abnormalities (3), poses a challenge to the use of traditional rehabilitation approaches for managing this condition. Further clarification of the relationships between HR and PA may help inform appropriate rehabilitation strategies for these patients. Therefore, the aim of this study was to examine the associations between HR (average daily HR and percentage of time in tachycardia) and PA (step count and accelerometer-defined PA intensity levels) in individuals with PCC, accounting for the presence of ME/CFS symptoms.

## METHODS

### Study design and participants

This observational study was approved by the research ethics committee of the University of Manitoba (HS25555) and conducted according to the Declaration of Helsinki. Following public advertising (social media and direct invitation to patients referred from outpatient rehabilitation clinics in Winnipeg, Canada), a convenience sample of 16 adults experiencing persistent symptoms for 3 months or more after acute COVID-19 infection was recruited (1). Those with PCC experiencing mild to severe respiratory symptoms and/or fatigue, and with access to a smart device and internet service at home were included. Exclusion criteria were acute lung or heart conditions, recent lung or cardiac surgery, severe hearing or visual impairments, inability to walk independently, high risk of falling and residence outside of Manitoba, Canada.

### Assessments and data collection

A total of 30 people indicated interest in participating in our study and were screened for eligibility via phone call. Twenty participants qualified; however, 1 participant dropped out and 3 were further excluded from the analysis due to incomplete watch data. The study was conducted in 2 phases between November 2022 and April 2023: an in-person assessment of approximately 2 h at the Respirability Lab, followed by home-based data collection. Informed consent was obtained from all participants before the assessment.

Participants self-reported their level of PA as either physically active or not physically active. Anthropometric data (height, weight, and body mass index) were collected at baseline, followed by dyspnoea (Modified Borg Scale, 0–10 points) (18) and fatigue (Fatigue Severity Scale). The latter consists of 9 items rated on a 7-point Likert scale ranging from 1 (strongly disagree) to 7 (strongly agree). The minimum and maximum scores obtainable are 9 and 63 respectively. The FSS also includes a visual analogue scale ranging from 0 (worst) to 10 (normal) to measure global fatigue (19).

The DePaul Symptom Questionnaire short-form (DSQ-SF) was applied to assess symptoms of ME/CFS by asking participants to rate the frequency and severity of 14 symptoms on a 5-point Likert scale (20). The presence of ME/CFS symptoms was defined according to the Canadian ME/CFS criteria, according to which participants should score at least 2 out of 5 in both severity and frequency across the domains of fatigue, post-exertional malaise, unrefreshing sleep, and neurocognitive impairment, and 1 symptom from at least 2 of the following domains: pain, autonomic, neuroendocrine, and immune (20). The total scores of each individual were averaged and rationalized to 100 points, and higher scores represented higher symptom burden.

Health-related quality of life was assessed via the EuroQol – 5 Dimensions – 5 Levels. This questionnaire consists of 2 parts: a visual analogue scale rated from 0 (worst imaginable health) to 100 (best imaginable health), and a section comprising 5 dimensions (mobility, self-care, usual activities, pain/discomfort, and anxiety/depression) rated from 1 (best) to 5 (worst). The total score for the VAS is 100, and each dimension is individually scored out of 5 (21).

The 1-minute sit-to-stand test (STST) and 6-minute walking test (6MWT) assessed exercise capacity. During the STST, participants were asked to stand upright and sit as many times as possible at a safe and comfortable pace for 1 min using a standard-height chair without armrests (22). The 6MWT involved the participants walking as far as possible along a 30-m track within 6 min (23). Resting periods were permitted during both tests, and the number of repetitions in the STST and the distance covered in the 6MWT were recorded and presented as absolute and percentage of predicted values (24, 25).

### Wearable devices

Participants were provided with a Garmin Venu^®^ SQ smartwatch (Garmin, Olathe, KS, USA) to continuously monitor HR. Garmin devices have high criterion validity and reliability (26). Participants were instructed to wear the smartwatch daily on their non-dominant wrist for 12 h during waking hours and synchronize their watch data in the Garmin Connect^®^ and Labfront apps (Labfront, Boston, MA, USA), which were previously installed on their smartphones during the in-person visit.

A hip-worn ActiGraph wGT3X-BT portable accelerometer (ActiGraph LLC, Pensacola, USA) was also given to the participants to track PA (according to counts per minute [cpm] and number of steps). The GT3X+ has also proven reliable for measuring PA in adults during everyday activities (27). Data were collected at 100 Hz and participants were instructed to wear the accelerometers for at least 12 h each day over 7 days. Wear time was also recorded in an activity log sheet provided to the participants for reference purposes during data processing. Non-wear times were defined as intervals of at least 60 consecutive minutes with zero activity counts. Ideally, a minimum wear time of 10 h/day for 4 days was expected for the accelerometer data to be included in analyses (28).

### Data processing and analysis

Accelerometer data were downloaded using ActiLife6 software and used to calculate PA levels according to the following cpm cut-off points: moderate to vigorous PA (MVPA) (> 760 cpm), light intensity PA (101 to 760 cpm), and sedentary (0 to 100 cpm) (29). Step-count was categorized according to the Tudor-Locke and Bassett (2004) classification as follows: sedentary lifestyle index (< 5,000 steps/day); low active (5,000 to 7,499 steps/day); somewhat active (7,500 to 9,999 steps/day); active (≥ 10,000 steps/day); and highly active (> 12,500 steps/day) (30). HR data from the Garmin smartwatches were matched minute by minute to the accelerometer data, and values above 100 bpm were considered tachycardia (31, 32). Average HR and percentage of minutes in tachycardia during sedentary time, light intensity time, and MVPA were calculated based on daily wear time.

### Statistical analysis

Data were presented as mean ± standard deviation. Group comparisons of individuals with and without ME/CFS symptoms were performed using unpaired *t*-test and the Mann–Whitney *U* test for continuous and ordinal data respectively. HR and PA data were analysed in 4-day clusters for all participants (including 1 participant with 3 days of data collected on the accelerometer). Generalized linear mixed models (fixed effects, linear distribution, and variance component matrix) were used to analyse the associations between PA (step count and cpm intensity levels) and HR (average HR and percentage of minutes in tachycardia). The crude model was adjusted for age, sex, and presence of ME/CFS symptoms (yes and no). Data were analysed using the SPSS software (IBM Corp, Armonk, NY, USA), and significance was set at *p* < 0.05.

## RESULTS

Sixteen individuals (13 females (81%), mean age of 51 ± 12 years) with PCC were included. Three participants (19%) were hospitalized during the acute COVID-19 illness and reported using respiratory equipment while being treated for the COVID-19 infection. All participants reported experiencing fatigue at the time of the initial assessment, while 14 (93%) reported shortness of breath, and 13 self-reported their PA level as not active. Overall, participants performed below the age- and sex-predicted values on the 6MWT (24) and 1-minute STST (25). Notably, the group with ME/CFS symptoms covered 27% less distance and completed 19% fewer repetitions than the group without these symptoms. Additionally, individuals with ME/CFS symptoms experienced greater difficulties in the EuroQoL domains of mobility, self-care, usual activities, and pain/discomfort (Table I).

**Table I:** Characteristics of study participants

Characteristic	All (*n* = 16)	Canadian ME/CFS criteria		
		Yes (*n* = 9)	No (*n* = 7)	*p*-value*
Age (years)	50.88 (11.97)	55.86 (9.44)	47.00 (12.78)	0.148
Height (m)	1.72 (0.94)	1.79 (0.9)	1.67 (0.06)	0.005
Body mass (kg)	86.60 (18.57)	87.4 (16.66)	85.94 (20.93)	0.883
BMI (kg/m^2^)	29.31 (6.52)	27.11 (3.86)	31.01 (7.82)	0.249
Modified Borg Scale (MBS)	2.03 (1.3)	2.5 (1.22)	1.43 (1.27)	0.110
FSS (total score)	54.75 (10.98)	59.22 (6.18)	49.00 (13.49)	0.062
FSS (VAS)	4.54 (2.91)	5.33 (3.39)	3.51 (1.94)	0.228
EuroQoL-5D-5L domains				
Mobility	2.19 (0.83)	2.56 (0.88)	1.71 (0.49)	0.040
Self-care	1.94 (0.93)	2.44 (0.88)	1.29 (0.49)	0.008
Usual activities	2.94 (1.06)	3.56 (0.88)	2.14 (0.69)	0.004
Pain/discomfort	2.44 (0.73)	2.89 (0.6)	1.86 (0.38)	< 0.001
Anxiety/depression	2.19 (1.05)	2.56 (1.13)	1.71 (0.76)	0.113
EuroQoL-5D-5L (VAS)	56.75 (17.39)	53.44 (16.82)	61.00 (18.48)	0.407
6MWT (metres)	344.02 (108.82)	271.56 (84.41)	437.19 (45.82)	< 0.001
6MWT (%pred)	61.67 (19.53)	49.92 (15.52)	76.76 (12.64)	0.002
1-minute STST (repetitions)	19.38 (6.49)	16.56 (6.48)	23.00 (4.69)	0.044
1-minute STST (%pred)	49.36 (16.81)	41.63 (16.05)	60.23 (8.74)	0.011
Average HR (beats per minute)	85 (6.22)	88 (5.80)	82 (4.50)	0.464
Percentage of time in tachycardia (%)	13.59 (5.70)	15.48 (15.58)	11.15 (5.99)	0.219
Step count	3503.06 (1445.23)	3402.78 (1544.29)	3632.00 (1416.85)	0.479

BMI: body mass index, m: metres, kg: kilogram, FSS (fatigue severity scale), 6MWT: 6-minute walk test, STST: Sit-to-stand test, pred: predicted, VAS: Visual Analogue Scale, EuroQol-5D-5L: EuroQol – 5 Dimensions – 5 Levels, MBS ([0 (no breathlessness] to 10 [worst]), FSS (0 [no fatigue] to 63 [worst]), FSS VAS (0 [worst] to 10 [normal]), EuroQol-5D-5L: EuroQol VAS (0 [worst health imaginable] to 100 [best health imaginable]; domains (1 [no problems] to 5 [worst]),

* p-value (between individuals with and without ME/CFS symptoms).

Participants wore the watches and accelerometers for a mean of 11.36 ± 2.60 and 12.51 ± 1.94 h per day, respectively. They walked 3,503.06 ± 1,445.23 steps per day, suggesting that they had low levels of PA and were classified as sedentary (30). No significant associations were observed between daily step count and average daily HR or percentage of time in tachycardia before and after adjusting for age, sex, and ME/CFS symptoms (Table II). Although higher PA levels according to the accelerometer data (cpm) were associated with increased average HR, participants displayed a significantly lower percentage of time in tachycardia during the MVPA level compared with the sedentary periods, even after adjusting for age, sex, and ME/CFS symptoms (Table III).

**Table II:** Associations between daily step count and HR parameters

Factor	Average HR			% time in tachycardia		
	β	CI (95%)	*p*-value	β	CI (95%)	*p*-value
Crude model						
Step count	–0.001	–0.001 to 0.001	0.813	0.001	0.000 to 0.001	0.860
Age and sex-adjusted						
Step count	–0.001	–0.001 to 0.001	0.799	0.001	0.000 to 0.001	0.842
Age	0.021	–0.227 to 0.269	0.868	–0.020	–0.105 to 3.316	0.653
Sex	2.952	–4.630 to 10.535	0.443	0.617	–2.083 to 3.316	0.635
Age, sex, and ME/CFS adjusted						
Step count	–0.001	–0.001 to 0.001	0.910	0.001	0.000 to 0.001	0.688
Age	0.089	–0.169 to 0.346	0.497	–0.009	–0.102 to 0.085	0.850
Sex	–0.495	–9.209 to 8.218	0.911	0.001	–3.292 to 3.294	0.999
ME/CFS symptoms (no)	Ref.					
ME/CFS symptoms (yes)	5.340	–1.847 to 12.526	0.144	0.926	–1.722 to 3.574	0.491

Individuals without ME/CFS symptoms were the category of reference. HR: heart rate, CI: confidence interval.

**Table III:** Associations between physical activity levels and HR parameters

Factor	Average HR			% time in tachycardia		
	β	CI (95%)	*p*-value	β	CI (95%)	*p*-value
Crude model						
PA levels						
Sedentary	Ref.					
Light PA	9.117	6.946 to 11.288	**< 0.001**	–1.516	–3.385 to 0.353	0.111
Moderate to vigorous PA	13.450	11.279 to 15.621	**< 0.001**	–3.380	–5.249 to –1.511	**< 0.001**
Age and sex adjusted						
PA levels						
Sedentary	Ref.					
Light PA	9.117	6.947 to 11.287	**< 0.001**	–1.516	–3.385 to 0.353	0.111
Moderate to vigorous PA	13.450	11.280 to 15.620	**< 0.001**	–3.380	–5.249 to –1.511	**< 0.001**
Age	0.019	–0.232 to 0.271	0.880	–0.020	–0.101 to 0.061	0.061
Sex	–3.030	–10.568 to 4.507	0.428	–0.677	–3.206 to 1.852	0.598
Age, sex, and ME/CFS adjusted						
PA levels						
Sedentary	Ref.					
Light PA	9.117	6.948 to 11.287	**< 0.001**	–1.516	–3.386 to 0.353	0.111
Moderate-to-vigorous PA	13.450	11.280 to 15.620	**< 0.001**	–3.380	–5.249 to –1.510	**< 0.001**
Age	0.091	–0.166 to 0.348	0.487	–0.009	–0.098 to 0.079	0.837
Sex	0.454	–8.026 to 8.934	0.916	–0.180	–3.167 to 2.807	0.905
ME/CFS symptoms (no)	Ref.					
ME/CFS symptoms (yes)	–5.497	–12.607 to 1.614	0.129	–0.829	–3.290 to1.633	0.507

Sedentary behaviour and individuals without ME/CFS symptoms were used as the category of reference. PA: physical activity, HR: heart rate, CI: confidence interval.

## DISCUSSION

To the best of our knowledge, this is the first study that aimed at clarifying the associations between tachycardia (a commonly reported symptom) and PA in individuals with PCC accounting for ME/CFS symptoms. The results of our study suggest that while HR increases with PA intensity level, the time spent in tachycardia is lower at the MVPA level than at the sedentary level. This finding aligns with complaints of “racing HR” commonly reported by PCC patients even during minimal or no activity (3). Our study provides valuable insights into the relationship between HR and PA, potentially guiding development of appropriate rehabilitation strategies for these patients.

The elevated HR experienced during sedentary periods by participants in our cohort can be attributed to autonomic dysfunction, presenting clinically as POTS or inappropriate sinus tachycardia (33). Physiological processes underlying this condition have been identified as downregulation of the renin–angiotensin–aldosterone system (RAAS), hyperadrenergic activity, and direct viral infection of the brainstem (34). This HR response is prevalent (35) and may be explained by episodic disability, which describes fluctuations in health that may lead patients to experience good and bad days related to their illness (36). In this sense, our participants may have chosen to avoid PA on bad days either due to poor health or to prevent the onset or worsening of symptoms and chosen to engage in MVPA on good days (i.e., when they experienced less severe tachycardia and other cardiovascular or respiratory symptoms) (3, 37). In addition, fear-avoidance behaviour, as seen in other post-viral conditions (39), could also have influenced this association (39).

Although both groups of our study participants experienced tachycardia at rest, individuals with PCC experiencing ME/CFS have shown greater fatigue and post-exertional malaise (40), symptoms that arise from physical or mental exertion. As a result, these individuals may avoid or decrease PA (41) to prevent symptom exacerbation. Although fear-avoidance behaviour may be effective in the short term, it may contribute to prolonged symptoms and deconditioning of the individual over time (39). Therefore, it is important to identify and address symptoms commonly experienced by individuals with PCC such as fatigue and dyspnoea (42), as they may contribute to reduce exercise capacity, limit daily activities (42), and hinder physical activity (43).

Similar to previous studies (44, 45), participants in our cohort demonstrated reduced exercise capacity, which can be attributed to skeletal muscle damage and weakness (46), mitochondrial myopathy (47), and deconditioning (48). Low PA was also identified in our participants, with 81.3% reporting themselves as inactive. The average daily step count recorded in our group by the wearable devices reflected a sedentary lifestyle (i.e., < 5,000 steps per day) (30), lower than previously observed in individuals with COPD (49) and interstitial lung diseases (50). Sedentary behaviour and low PA levels may pose long-term health risks because they are independent factors of cardiovascular disease, reduced quality of life, and all-cause mortality (51, 52). Therefore, these factors should be addressed in PCC individuals.

While rehabilitation may help counteract deconditioning and lack of PA, caution is necessary when incorporating exercise into rehabilitation programmes with these patients. Skeletal muscle mitochondria changes, inflammation, and capillary injury may contribute to fatigue (53), and physical activity may further aggravate these processes (54); therefore, a safe and gradual increase in PA should be encouraged to avoid symptom exacerbation. The use of individualized and supervised exercise programmes (55), together with pacing during PA (56), may help mitigate worsening of symptoms. Additionally, HR should be closely monitored during PA, as abnormal HR responses and cardiovascular autonomic dysfunction have been found in this population (33). In our cohort, participants spent significantly more time in tachycardia while sedentary than during MVPA, further supporting this concern. As shown in our study, monitoring can be done using commercial wearable devices, particularly smartwatches, which are accessible tools to achieve this purpose. Wearable HR data can offer meaningful insight into cardiovascular function in this population. Time spent in tachycardia, especially during periods of sedentary activity, may indicate autonomic dysfunction or cardiovascular strain (5). Measures such as resting HR trends, HR variability, and HR recovery after activity may further reflect impaired autonomic regulation or deconditioning (57). Monitoring HR responses during PA also helps identify chronotropic abnormalities (58). Collectively, these indicators support safer exercise prescription, early detection of cardiovascular stress, and individualized rehabilitation planning.

Some limitations should be considered when interpreting our study results as they may constrain the general applicability of our findings. First, the final sample size was smaller than anticipated, which may impact on the generalizability of the findings. Several factors may have contributed to this, including limited symptom recognition among patients and healthcare providers, as well as the lack of a dedicated PCC clinic and centralized referral pathways, which together may have hindered the identification and recruitment of eligible participants. It is also unclear whether the limited sample size was due to participants’ inability or unwillingness to participate. Despite multiple recruitment efforts, only 30 individuals expressed interest, with 20 meeting the inclusion criteria. One participant withdrew due to financial hardship related to illness, and 3 were excluded from analysis due to incomplete data resulting from difficulty adhering to the study protocol. Second, identifying awake/active times during device wear times was not straightforward for all participants due to the variability in individual schedules. However, information from the device and the participants’ logs ensured accurate sorting of the information. Also, HR variability was not assessed, nor was a control group included, which may have better identified unique responses to PA in our PCC cohort.

This study provides valuable insights concerning HR responses to PA in individuals with PCC. Although ME/CFS symptomatic participants in our cohort exhibited poorer performance on physical function tests, potentially reflecting lower physical capacity and possibly contributing to reduced engagement in regular physical activity, this factor did not influence the associations between HR and PA in our study. Furthermore, the use of wearable devices for data collection in this study provided a means for assessing and monitoring outcomes in PCC, which may facilitate patient self-management and rehabilitation in this population. Additional research should examine the relationship between PCC symptoms, tachycardia, and PA while also exploring the impacts of dysautonomia on the PA of individuals with PCC. Future studies should aim for larger and more balanced sample sizes to increase statistical power, enhance generalizability, and enable detailed subgroup analyses. Additionally, collecting 24-h data would allow for a clearer distinction between PA, sedentary behaviour, and sleep, providing a comprehensive view of daily patterns and circadian rhythms.

### Conclusion

Individuals with PCC in our study experienced more tachycardia while doing little to no activity than while engaged at higher intensity PA levels independently of the presence of ME/CFS symptoms. This finding is consistent with reports commonly made by PCC patients and provides valuable insights into the association between HR and PA, which may be useful for informing patient care and future studies.
